# Natural and Synthetic Peptides as Alternatives to Antibiotics in Intestinal Infections—A Review

**DOI:** 10.3390/antibiotics15010068

**Published:** 2026-01-08

**Authors:** Lala Stepanyan, Monika Israyelyan, Alessandro Gori, Avetis Tsaturyan, Zhaklina Saribekyan, Kristina Hovsepyan, Tatevik Sargsyan, Raffaele Pastore, Antonio De Luca, Giovanni N. Roviello

**Affiliations:** 1 Scientific and Production Center “Armbiotechnology” NAS RA, 14 Gyurjyan Str., Yerevan 0056, Armenia; 2 SCITEC—Istituto di Scienze e Tecnologie Chimiche “Giulio Natta”, National Research Council of Italy (CNR), Via Mario Bianco 9, 20131 Milano, Italy; 3 Institute of Pharmacy, Yerevan State University, 1 Alex Manoogian Str., Yerevan 0025, Armenia; 4 Department of Medicine and Health Sciences Vincenzo Tiberio, University of Molise, Via F. De Santis, 86100 Campobasso, Italy; 5 Department of Mental and Physical Health and Preventive Medicine, Section of Human Anatomy, University of Campania “Luigi Vanvitelli”, Via Luciano Armanni, 80138 Naples, Italy; 6 Institute of Biostructures and Bioimaging, National Research Council of Italy (CNR), Via Tommaso De Amicis 95, 80145 Naples, Italy

**Keywords:** intestinal infections, antimicrobial peptides, gut microbiota, epithelial barrier, defensins, cathelicidins, synthetic antimicrobial peptides, antibiotic resistance, anti-infective therapy

## Abstract

Background/Objectives: Antimicrobial peptides (AMPs), evolutionarily conserved components of innate immunity characterized by their broad-spectrum efficacy and minimal resistance development, are increasingly recognized as promising therapeutic candidates. This review aims to integrate current knowledge concerning natural and synthetic antimicrobial peptides and their therapeutic effectiveness in addressing gastrointestinal infections. Methods: A literature review was performed, evaluating recent peer-reviewed studies on AMPs. The research concentrated on their molecular mechanisms of action, antimicrobial spectrum, and their interactions with standard antibiotics. More in detail, the peptide classes examined herein included defensins, cathelicidins, histatins, and various natural peptides such as lactoferricin, protamines, RegIII, and hepcidin, along with synthetic analogs like WR12, D-IK8, MSI-78, and IMX942. Results: Natural AMPs demonstrated significant antimicrobial and immunomodulatory effects against *Escherichia coli*, *Klebsiella pneumoniae*, *Salmonella* spp., and *Shigella* spp. Beyond direct antimicrobial activity, antimicrobial peptides act as integrated anti-infective agents not only by modulating host–microbiota interactions, but also preserving epithelial barrier integrity, and limiting inflammation, thereby offering a multifaceted strategy to control gastrointestinal infections. On the other hand, synthetic peptides showed improved stability, reduced cytotoxicity, and synergistic interactions with antibiotics, which suggests that they could be used either alone or in combination with other treatments. Conclusions: AMPs constitute a promising category endowed with anti-infective activity, especially for therapy of intestinal diseases, which is attributed to their distinctive anti-infective mechanisms, immune-modulating characteristics, and a relatively low propensity for resistance development compared to conventional antibiotics. However, more clinical trials and improvements to their formulation are needed to translate promising in vitro results into reliable patient outcomes.

## 1. Introduction

Intestinal infections remain a pressing global health challenge, largely due to their high prevalence and the growing resistance of pathogenic microorganisms to existing antimicrobial agents [1,2,3,4]. In recent years, several Gram-negative and Gram-positive pathogens, including *Escherichia coli*, *Klebsiella pneumoniae*, *Salmonella* spp., and *Shigella* spp., have developed multidrug-resistant (MDR) and even extremely drug-resistant (XDR) traits [5,6,7,8]. Consequently, the efficacy of traditional antibiotics has markedly declined. This issue is particularly pronounced in developing countries, where inappropriate antibiotic use and inadequate regulatory enforcement accelerate the dissemination of resistant strains. The resulting increase in morbidity and mortality highlights the urgent need for innovative therapeutic approaches and strengthened public health measures [9,10,11]. With the declining efficacy of conventional antibiotics, antimicrobial peptides (AMPs) have emerged as a promising alternative. AMPs are naturally occurring molecules present across nearly all forms of life and constitute a fundamental component of the innate immune system. Beyond their ability to disrupt bacterial membranes, they also modulate host inflammatory responses, with high concentrations of these peptides being found within the intestinal mucosa, a critical protective barrier of the body. Produced primarily by epithelial and Paneth cells, AMPs prevent pathogen adhesion and invasion while simultaneously contributing to the maintenance of intestinal microbiota homeostasis [12,13,14,15,16,17]. Over the past few years, significant progress has been achieved in developing synthetic peptides that can overcome the limitations of natural antibacterial peptides, such as lower stability or reduced activity under normal conditions. In this regard, structural modifications, amino acid substitutions, and modern design methodologies enabled the production of more stable, targeted, and effective peptides, thereby increasing their potential for treating intestinal infections [16,17,18,19,20]. The objective of this review is to provide a comprehensive overview of the current knowledge on both natural and synthetic antimicrobial peptides, with particular emphasis on their biological functions, mechanisms of action, and potential clinical applications as anti-infective agents. In fact, as we discuss below, antimicrobial peptides are increasingly regarded as safe and effective candidates for next-generation antibiotics, offering promising strategies to combat intestinal infections and address the escalating global crisis of antibiotic resistance.

### Methods

To ensure a comprehensive analysis of the role of antimicrobial peptides in intestinal infections, a literature search was conducted. The search was performed using different databases including Google Scholar, PubMed/MEDLINE, Scopus, and Web of Science. The search covered the period from January 2002 to December 2025, with a special focus on studies published in the last 5 years to ensure the relevance of the data. The following keywords and their combinations were used: “antimicrobial peptides”, “AMPs”, “intestinal infections”, “gut microbiota”, “defensins”, “cathelicidins”, “LL-37”, “synthetic peptides”, “antibiotic resistance”, “gastrointestinal tract”, and “peptide engineering”. The inclusion criteria were original research articles and reviews published in English; studies focusing on the mechanism of action, immunomodulatory effects, and clinical potential of natural and synthetic AMPs in the context of intestinal infections; and papers describing interactions between AMPs and common intestinal pathogens (*Escherichia coli*, *Salmonella* spp., *Shigella* spp., *Klebsiella pneumoniae*). The exclusion criteria were papers without full-text availability, studies focusing solely on non-intestinal infections (e.g., respiratory or skin infections) unless relevant mechanistic parallels were drawn, and duplicate publications.

Study quality and relevance were evaluated using predefined criteria, including clarity of experimental design, appropriateness of methods used to assess antimicrobial or immunomodulatory activity, relevance to intestinal pathogens or gut immunity, and robustness of reported outcomes. This process was guided by general principles of scientific literature analysis and informed by elements of the PRISMA framework. As for data selection and analysis, initially, a total of 800 records were identified through database searching. After removing duplicates, identified through visual inspection and verification, and screening titles/abstracts for relevance, 250 full-text articles were assessed for eligibility. Finally, 216 studies were selected for inclusion in this review.

## 2. AMPs as Emerging Therapeutics for Gastrointestinal Pathogens

This review synthesizes current scientific evidence on the structural organization, biological activities, and therapeutic potential of both natural and synthetic AMPs in the prevention and treatment of gastrointestinal infections [14,21,22]. The studies reviewed elucidate not only the molecular mechanisms of action of AMPs but also features such as their spectrum of antimicrobial activity, synergism with conventional antibiotics, as well as their role in regulating intestinal immunity and preserving the epithelial barrier [23,24,25]. The major classes of natural antimicrobial peptides, such as defensins, cathelicidins, histatins, lactoferricin, protamines, members of the RegIII family, and hepcidin, exhibit potent antimicrobial, anti-inflammatory, and immunomodulatory activities against clinically relevant pathogens, including *Escherichia coli*, *Klebsiella pneumoniae*, *Salmonella* spp., and *Shigella* spp. [26,27,28,29]. On the other hand, synthetic analogues developed through rational peptide-engineering strategies exhibit enhanced physicochemical and functional characteristics relative to natural AMPs and can be specifically designed to achieve increased proteolytic stability together with potentially reduced cytotoxicity and improved biocompatibility [30,31,32,33]. Table 1 presents a list of bioactive antimicrobial peptides currently under investigation at different stages of biological activity research.

Interestingly, multiple studies have observed synergistic effects between synthetic AMPs and antibiotics, thereby enhancing the prospects for combinatorial strategies in the management of antibiotic-resistant infections. The available evidence supports the classification of antimicrobial peptides as a distinct category of biomolecules with considerable clinical potential, owing to their broad spectrum of activity, lower propensity for resistance development when compared to other antibiotics, and ability to maintain the integrity of the intestinal epithelial barrier [85,86,87,88]. These findings suggest the necessity for additional research focused on enhancing their pharmaceutical formulations, together with improving AMP stability, and mitigating the potential toxicity observed in some cases. Understanding these aspects of AMPs is crucial, especially in considering their interactions with the gut microbiota, which will be discussed in the next section.

### 2.1. Interaction Between Intestinal Microbiota and Epithelial Antimicrobial Peptides

The intestinal microbiota represents a highly organized consortium of microorganisms, predominantly comprising members of the phyla *Bacteroidetes*, *Firmicutes*, *Actinobacteria*, and *Proteobacteria*. These bacterial groups play essential roles in host metabolism, regulation of the immune system, defense against pathogenic organisms, and maintenance of intestinal barrier integrity [89,90,91]. Epithelial-derived AMPs, produced primarily by Paneth cells and enterocytes along the crypt–villous axis, constitute a critical component of the gut’s innate defense system. These peptides regulate the dynamic interactions between the intestinal microbiota and epithelial cells. Their functions extend well beyond direct antimicrobial activity and include the following:

(i) Maintaining the spatial structure of microbial communities, forming distinct gastrointestinal niches for various taxa;
(ii) Preventing colonization by pathogens by limiting their adhesion and invasion;
(iii) Creating a sterile layer over the epithelium (especially due to RegIIIγ), preventing bacterial contact with the mucosa;
(iv) Regulating innate immunity, including modulating Toll-like receptors, producing cytokines, and controlling the inflammatory response.

Epithelial antimicrobial peptides work together to control the microbiota on many levels. They achieve this by directly eliminating microorganisms, fine-tuning the immune response, and keeping the barrier intact. However, the contribution of individual classes of AMPs to these processes varies and is determined by their structural and functional properties. Defensins, the most extensively characterized group of epithelial AMPs, occupy a particularly prominent role. They have been shown to play a key role in crypt protection, regulation of microbiota composition, and are implicated in the pathogenesis of inflammatory bowel diseases, most notably ileal Crohn’s disease. Accordingly, a detailed examination of the biological properties and mechanisms of action of defensins is warranted, as they represent the principal effector molecules of innate intestinal immunity [92,93,94,95,96,97]. Thus, the anti-infective efficacy of intestinal antimicrobial peptides is tightly linked to their ability to regulate host–microbiota interactions, thereby limiting pathogen overgrowth while maintaining a balanced and protective microbial ecosystem in the gut.

#### 2.1.1. Defensins as Key Epithelial Effector Molecules of Intestinal Innate Immunity

**Figure 1:**
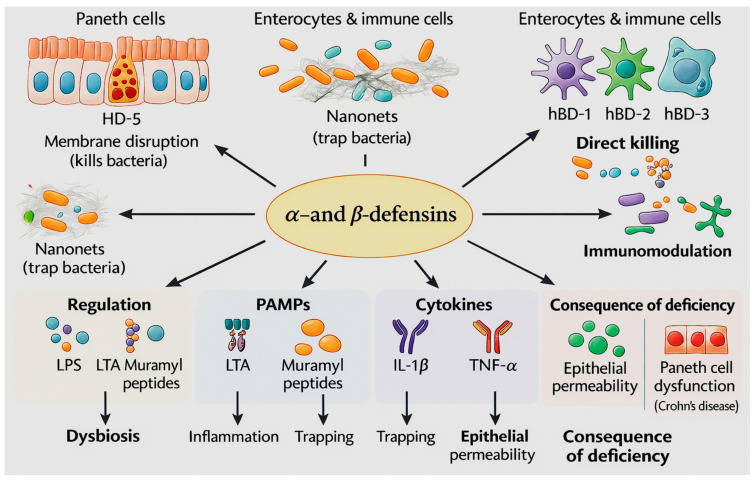
Intestinal defensins: sources, molecular mechanisms, regulation, and pathological consequences. The benefits of AMP activity are effective bacterial killing through membrane disruption, trapping of microbes via nanonets, modulation of immune signals, and maintenance of epithelial integrity and microbiota balance. The risks associated with AMP deficiency are increased epithelial permeability, dysbiosis, heightened inflammation driven by PAMPs and cytokines, and Paneth cell dysfunction linked to conditions such as Crohn’s disease.

As anticipated, defensins are the most studied and important group of epithelial antimicrobial peptides, playing a crucial role in maintaining gut homeostasis. They directly eliminate bacteria, control the arrangement of the microbiota, and prevent pathogens from adhering to and penetrating the intestinal epithelium. The human intestine contains two main types of defensins: α-defensins, which are produced by Paneth cells, and β-defensins, synthesized by enterocytes and mucosal immune cells [98,99]. Figure 1 (below) provides an overview of the cellular origins, antimicrobial mechanisms, and immunological functions of intestinal α- and β-defensins. These peptides, produced by Paneth cells, enterocytes, and mucosal immune cells, are discussed in greater detail below.

Paneth cells represent the principal source of α-defensins in the small intestine, producing human defensin-5 (HD-5) and human defensin-6 (HD-6). These peptides display potent activity against a wide range of Gram-negative and Gram-positive bacteria, including clinically relevant enteropathogens such as *Escherichia coli*, *Salmonella* spp., *Klebsiella pneumoniae*, and *Shigella* spp., including multidrug-resistant strains. HD-5 exhibits a potent direct bactericidal effect by disrupting the integrity of bacterial membranes. HD-6, in contrast, acts in a non-standard manner: it forms amyloid-like fibrils that form so-called “nanowebs” that physically trap bacteria and prevent them from penetrating the crypts. This mechanism ensures control of microbiota without excessive inflammation, which is critical for maintaining epithelial integrity [100,101,102,103]. β-Defensins (hBD-1, hBD-2, hBD-3) are produced primarily by enterocytes and immune system cells (macrophages, dendritic cells) in response to bacterial pathogen-associated molecular pattern (PAMP) molecules: lipopolysaccharide (LPS), lipoteichoic acid, and muramyl peptide derivatives, as well as proinflammatory cytokines (IL-1β, TNF-α). The functional distribution of β-defensins reflects their diverse roles in intestinal immunity. In particular, hBD-1 is a constitutively expressed peptide that provides a basic level of protection; in contrast, hBD-2 is induced during inflammation and has pronounced activity against Gram-negative bacteria, while hBD-3 exhibits a broad spectrum of antimicrobial activity, including effectiveness against yeast fungi (*Candida* spp.). The balance of α- and β-defensins is critical for homeostasis, with a deficiency in their secretion being closely associated with intestinal diseases. For instance, in ileal Crohn’s disease, reduced α-defensin levels arise from Paneth cell dysfunction, while diminished β-defensin expression is linked to dysbiosis, chronic inflammation, and increased epithelial permeability. Collectively, defensins constitute essential components of the innate intestinal immune system, providing direct antimicrobial activity, reinforcing the mucosal barrier, and regulating the composition of the gut microbiota. Their deficiency results in barrier dysfunction, pathogenic alteration of the microbiome, and heightened vulnerability to inflammatory diseases [104,105,106,107]. Overall, defensins constitute a first-line anti-infective barrier in the intestine, exerting potent activity against enteric pathogens while shaping microbiota composition and preventing bacterial invasion of the epithelial surface. However, therapeutic manipulation of defense peptides remains challenging due to their cell-type specificity [108], the theoretical risk of microbiota modification, and the limited number of clinical studies validating their safety and efficacy. Although endogenous defensins appear to support commensal colonization and contribute to microbiota recovery after dysbiosis [109], further research is needed to clarify how exogenous defensins or defensin-based therapeutics interact with the intestinal ecosystem when administered in a therapeutic context.

#### 2.1.2. Cathelicidin LL-37 in the Gut: Immunomodulatory Mechanisms and Barrier-Protective Functions

**Figure 2:**
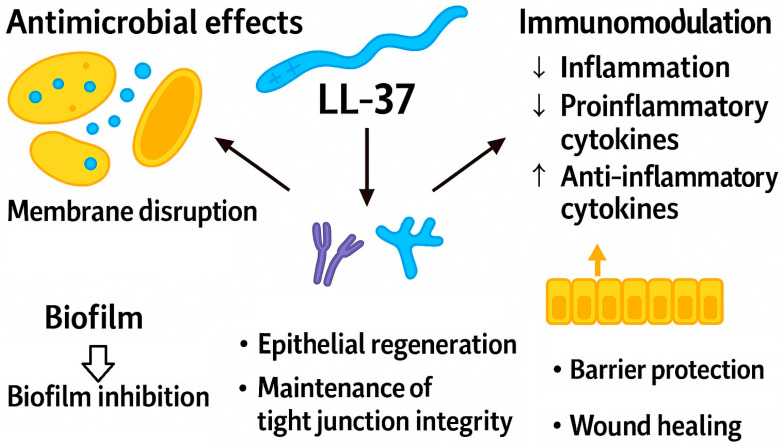
Illustrative overview of the antimicrobial, immunomodulatory, and barrier-protective functions of LL-37.

Cathelicidins are epithelial antimicrobial peptides with both antimicrobial and immunomodulatory functions. In humans, the sole representative is LL-37, an amphiphilic α-helical peptide. LL-37 exerts broad activity against intestinal pathogens such as *Escherichia coli*, *Salmonella* spp., *Shigella* spp., and *Klebsiella pneumoniae* by destabilizing bacterial membranes, forming pores, and preventing biofilm formation. It also blocks adherence and invasion of enteropathogenic *E. coli*, thereby protecting epithelial cells. Importantly, LL-37 displays relatively increased resistance to proteolytic degradation compared to many other natural antimicrobial peptides, allowing it to retain activity in the intestinal environment. Figure 2 illustrates LL-37’s principal activities, including membrane disruption, biofilm inhibition, modulation of inflammatory signaling, and promotion of epithelial repair and tight-junction integrity [110,111].

The most critical property of LL-37 is its ability to modulate the intensity of the inflammatory response, thereby preventing excessive immune activation. This peptide binds to and neutralizes LPS, inhibiting Toll-like receptor 4 (TLR4)-mediated initiation of the inflammatory cascade. LL-37 further suppresses NF-κB- and MAPK-dependent signaling pathways, resulting in reduced production of proinflammatory cytokines (TNF-α, IL-6, IL-1β) and enhanced secretion of the anti-inflammatory cytokine IL-10. In addition, LL-37 influences the functional activity of macrophages, neutrophils, and dendritic cells. These properties render LL-37 particularly significant in intestinal disorders characterized by excessive inflammation, including infectious colitis and inflammatory bowel disease [112,113,114]. In addition to its bactericidal activity, LL-37 promotes epithelial cell migration, proliferation, and differentiation. It accelerates wound healing and facilitates the restoration of mucosal tissue, while also reinforcing tight junctions between epithelial cells. These actions collectively enhance barrier integrity and prevent the translocation of bacteria and toxins across the intestinal epithelium. In models of infection induced by *Citrobacter rodentium* or enterohemorrhagic *Escherichia coli* (EHEC), LL-37 diminishes epithelial damage, reduces neutrophil infiltration, and expedites barrier restoration. Evidence shows that LL-37 acts as both an antimicrobial molecule and a regulator of immune homeostasis. LL-37 prevents excessive inflammation, preserves epithelial barrier integrity, and accelerates the healing of epithelial tissue following infection. Owing to its ability to modulate diverse immune and regenerative processes, LL-37 is regarded as one of the most versatile and multifunctional antimicrobial peptides in the intestine [115,116,117,118]. Thus, LL-37 plays a dual anti-infective role in the intestine by directly eliminating enteric pathogens and modulating inflammatory responses, thereby limiting tissue damage during intestinal infections. From a translational and therapeutic standpoint, future research must address key clinical limitations of LL-37, including its potential cytotoxicity at elevated concentrations, biochemical instability, and dose-dependent constraints. Moreover, overcoming challenges related to oral or luminal delivery remains essential for its effective application in gastrointestinal settings.

#### 2.1.3. Regenerating AMPs (RegIII) and the Spatial Organization of the Gut Microbiota

**Figure 3:**
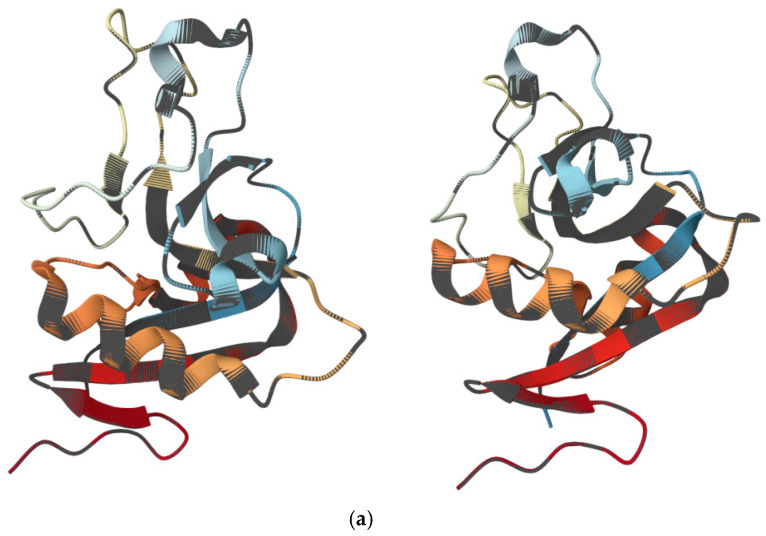

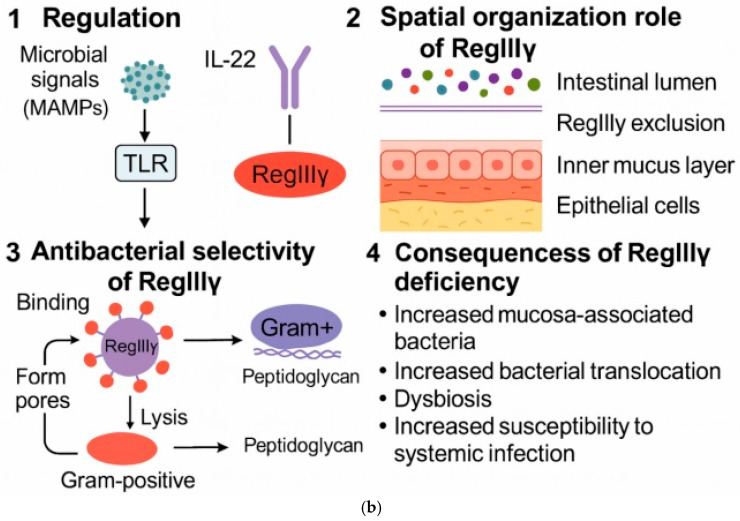
(**a**) Three-dimensional views of RegIIIα, one of the members of the RegIII family, shown from different orientations. The structure was visualized and rotated using the 3D viewer available at the Protein Data Bank (https://www.rcsb.org/3d-view/4MTH/1, accessed on 11 December 2025). (**b**) Scheme illustrating the key functions and regulatory mechanisms of RegIIIγ.

The RegIII family (Figure 3a) of regenerating-like lectins represents a distinct class of intestinal epithelial AMPs. In contrast to defensins or LL-37, which primarily eliminate microorganisms by disrupting their membranes, RegIII peptides contribute mainly to the spatial organization of the gut microbiota, preserving physical separation between commensal bacteria and host tissues (Figure 3b). This function is increasingly recognized as a key element of mucosal homeostasis [119,120]. Regarding structural features and regulation, RegIIIγ, the best-characterized member of the regenerating gene family in mice, is a C-type lectin-like antimicrobial peptide produced by Paneth cells and enterocytes [121,122]. Its expression is tightly regulated by microbial and immune signals. Microbial-associated molecular patterns trigger TLR/MyD88-dependent signaling in intestinal epithelial cells, resulting in the induction of RegIIIγ expression in response to bacterial colonization. In addition, IL-22, produced predominantly by type 3 innate lymphoid cells (ILC3s) and Th17 cells, markedly upregulates RegIIIγ expression during increased microbial load, inflammation, or infection. This dual regulation enables epithelial cells to rapidly adjust their antimicrobial activity according to environmental conditions. RegIIIγ binds exposed peptidoglycan on the surface of Gram-positive bacteria, which underlies its selective antimicrobial spectrum [123,124]. With respect to spatial barrier formation, one of the most critical functions of RegIIIγ is the maintenance of a bacteria-free exclusion zone immediately above the intestinal epithelium. This antimicrobial lectin thereby enforces a physical separation between commensal microbiota and host tissues, preserving epithelial integrity and preventing inappropriate immune activation. Thus, rather than acting solely as a classical membrane-disrupting AMP, RegIIIγ primarily shapes the spatial structure of the microbiota in vivo [119,121]. It helps to prevent direct microbial contact with the apical surface of enterocytes, restricts colonization of the mucosal surface by commensals, and limits the expansion of mucosa-associated bacteria. In various experimental models, deficiency of RegIII lectins leads to increased mucosa-associated bacteria, enhanced bacterial translocation to mesenteric lymph nodes and liver, as well as a higher risk of systemic complications. In other words, RegIIIγ acts as a chemical barrier that maintains a safe distance between the host and its dense microbial community [119,121].

As for the mechanisms of antibacterial activity, RegIIIγ primarily enforces spatial segregation of bacteria from the mucosal surface but also displays direct bactericidal activity against Gram-positive species. It binds peptidoglycan, oligomerizes on the bacterial surface, and forms pore-like structures that disrupt membranes and cause lysis. Gram-negative bacteria are less susceptible due to their protective outer membrane. Beyond antimicrobial effects, RegIIIγ supports intestinal homeostasis by stratifying microbial communities, preserving barrier integrity during inflammation, and limiting bacterial translocation. Reduced expression is linked to dysbiosis, inflammatory bowel disease (IBD), and impaired host–microbiota balance [119,121,125,126]. Overall, RegIII antimicrobial peptides contribute to intestinal anti-infective defense primarily by enforcing spatial segregation between bacteria and the epithelium, reducing pathogen translocation, and preventing infection-driven inflammation.

#### 2.1.4. The Overall Role of Epithelial Antimicrobial Peptides in Intestinal Homeostasis

Remarkably, epithelial antimicrobial peptides form a multi-level biochemical defense system, providing both direct antimicrobial protection and regulating interactions between the epithelium and microbiota [127,128]. Their coordinated activity encompasses several complementary functions: direct suppression of pathogen growth through membrane disruption, biofilm inhibition, and toxin neutralization. Other functions comprise the stabilization and spatial organization of the commensal microbiota, including control of opportunistic bacterial expansion, the modulation of the inflammatory response to prevent excessive immune activation and tissue damage, and the enhancement of mucosal barrier function by strengthening intercellular junctions and preventing bacterial translocation across the epithelium [129,130,131]. Two-way interactions with microbial metabolites, such as short-chain fatty acids and indole derivatives, tightly regulate AMP expression and contribute to the maintenance of the immune tone. Consequently, epithelial AMPs serve as a central link in sustaining a stable bidirectional contact between the host and its microbiota. They ensure a delicate balance between antimicrobial defense, regulation of inflammation, and preservation of intestinal barrier integrity. Impaired AMP secretion disrupts this equilibrium, leading to dysbiosis, heightened susceptibility to infections, and the development of inflammatory bowel diseases, demonstrating their fundamental importance for intestinal homeostasis [127,128]. Although intestinal AMPs, such as defensins, cathelicidins, and Reg III, are endowed with unique functions in the mucosa, they represent only part of a much larger family of natural antimicrobial peptides found in various organisms and sharing common structural motifs. Understanding the universal structural classes of natural AMPs, regardless of their tissue origin, not only explains the similarities in their mechanisms of action but also substantiates their therapeutic potential, ultimately serving as the foundation for the development of synthetic analogues [132,133,134]. Hence, through coordinated antimicrobial, immunomodulatory, and barrier-protective activities, epithelial antimicrobial peptides form an integrated anti-infective system that safeguards intestinal homeostasis during microbial challenge.

### 2.2. Natural Antimicrobial Peptides: Structural Organization and Mechanisms of Action

Most natural AMPs are a heterogeneous group of low-molecular-weight cationic peptides that constitute the primary chemical barrier at mucosal surfaces. All natural AMPs share key physicochemical traits, such as a positive charge, amphipathicity, and affinity for negatively charged microbial lipids, despite differing in origin, sequence, and length. This conserved structure provides a rapid, evolutionarily stable defense that limits resistance and makes AMPs essential for intestinal protection against infection [135,136,137]. The conserved structural features of natural antimicrobial peptides underpin their rapid and broad anti-infective activity in the intestine, enabling effective control of enteric pathogens with a reduced risk of resistance development.

#### 2.2.1. Structural Diversity of Natural AMPs

The structural features of AMPs determine the mechanism of their interaction with membranes and the spectrum of their antimicrobial activity. Four architectural classes are most common: α-helical peptides, β-sheet structures, extended (linear) peptides, and α/β-combined molecules. Interestingly, α-helical AMPs such as LL-37 and lactoferricin lack disulfide bonds and display strong amphipathicity. Due to their flexibility and ability to assume a helical shape only upon contact with the membrane, these peptides effectively integrate into the lipid bilayer and form pores, causing rapid osmotic lysis of bacteria. In this context, in addition to its direct antimicrobial function, LL-37 regulates epithelial regeneration, downregulates proinflammatory signaling pathways, and neutralizes endotoxins, making it a unique link between antimicrobial defense and immunomodulation [133,138,139]. On the other hand, β-sheet AMPs, which include α- and β-defensins, are characterized by a stable conformation reinforced by disulfide bridges. The structural configuration of defensins confers high resistance to proteolysis, a property particularly important in the intestinal lumen, which is rich in digestive enzymes and microbial proteases. Defensins exert broad-spectrum antimicrobial activity against Gram-positive and Gram-negative bacteria, including multidrug-resistant strains, as well as yeasts. Beyond their bactericidal capacity, defensins also contribute to barrier formation; for example, HD-6 assembles into ‘nanonets’ that physically entrap pathogens, preventing their access to intestinal crypts without eliciting excessive inflammation. In contrast, long linear AMPs such as indolicidin, histatin-5, and tritrpticin lack a defined secondary structure and are enriched in aromatic and cationic amino acids. This structural flexibility enables them to insert into membranes through multiple pathways and disrupt target cell function. Owing to their ability to penetrate bacterial cells, linear AMPs frequently inhibit DNA and protein synthesis, thereby functioning in a manner that parallels traditional chemotherapeutic agents [133,138,139]. Combined α/β structures, including protegrins and thanatin, are compact, disulfide-stabilized molecules that combine mechanical stability and high activity. These peptides exhibit pronounced bactericidal activity and the ability to disrupt biofilms, which is particularly important in the treatment of chronic infections [138,139,140]. Thus, the structural diversity of natural antimicrobial peptides determines their spectrum and mode of anti-infective action in the gut, allowing for efficient targeting of intestinal pathogens through membrane disruption and intracellular interference.

#### 2.2.2. Molecular Mechanisms of Action of Natural AMPs

**Figure 4:**
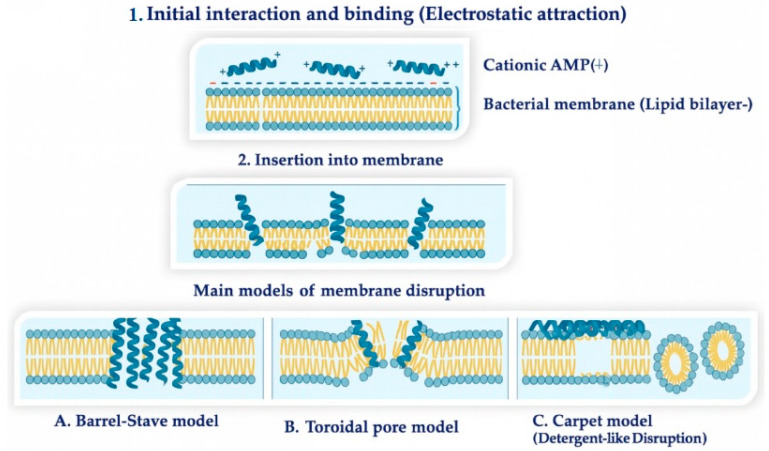
Schematic overview of the main mechanisms of membrane disruption adopted by antimicrobial peptides.

Owing to the physicochemical characteristics of bacterial membranes, their lipid composition is a crucial determinant of membrane behavior and cellular responses. Different bacterial species possess distinct membrane profiles, and even within a single species, the membrane makeup is not fixed but adapts to environmental conditions. Bacterial membranes contain a wide array of amphiphilic lipids. Among the most common are phosphatidylglycerol, phosphatidylethanolamine and cardiolipin, while phosphatidylcholine and phosphatidylinositol appear less frequently. In addition to these phospholipids, bacteria may incorporate other lipid classes such as ornithine lipids, glycolipids, sphingolipids, hopanoids, and various specialized membrane components [141]. Antimicrobial peptides typically exert their bactericidal effect through direct contact with the cell membranes of pathogens. AMPs interact with negatively charged lipids to trigger a sequence of structural changes that disrupt the integrity of membranes (Figure 4). In this frame, the barrel-stave, toroidal pore, and carpet-like models are the most studied mechanisms describing how AMPs eliminate both Gram-positive and Gram-negative bacteria. Clinically, AMPs perform better when modified with synthetic strategies such as D-amino acids, lipidation, and cyclization, which stabilize pore formation. The process begins with cationic AMPs binding via electrostatic forces to bacterial surfaces, lipopolysaccharides in Gram-negative and peptidoglycan in Gram-positive species, followed by insertion into the lipid bilayer that destabilizes membranes. The final step involves pore formation: cylindrical pores (barrel-stave), toroidal pores (toroidal pore), or carpet-like disintegration, all leading to cell death. These mechanisms apply to both natural and synthetic AMPs, ensuring rapid bacterial clearance. Beyond direct bactericidal activity, natural AMPs interact with host immunological receptors, inhibit Toll-like receptor signaling, reduce proinflammatory cytokines, and protect mucosal tissue, uniquely combining antimicrobial and anti-inflammatory functions [17,133,142,143].

Recent data show that natural AMPs are a promising basis for intestinal therapeutics, being effective against *Escherichia coli*, *Shigella* spp., *Salmonella* spp., and *Klebsiella pneumoniae*, including multidrug-resistant strains, while protecting and accelerating healing of epithelial cells, blocking pathogen attachment and invasion, and preventing bacterial translocation into the bloodstream and systemic disease. By altering fundamental membrane physicochemical properties, they reduce the likelihood of resistance development, thus suggesting their clinical potential; however, instability, rapid proteolysis, and low bioavailability limit their use, leading to the creation of synthetic analogues and peptidomimetics that retain antimicrobial efficacy but exhibit superior pharmacological properties [17,133,144,145]. Overall, by targeting fundamental membrane properties and key immune signaling pathways, natural antimicrobial peptides exert rapid anti-infective effects against intestinal pathogens while simultaneously limiting inflammatory tissue damage.

### 2.3. Synthetic Antimicrobial Peptides: A Modern Idea and Their Role in Medicine

Synthetic AMPs represent a robust class of molecules designed to mimic natural peptides of the innate immune system while exhibiting improved drug-like properties. They are regarded as promising alternatives to conventional antibiotics due to their potent bactericidal activity, their ability to disrupt biofilms, and their broad-spectrum efficacy against diverse pathogens, coupled with a reduced propensity for resistance development. Advances in peptide engineering have further enhanced their therapeutic potential by improving stability, bioavailability, and safety through strategies such as incorporation of D-amino acids, cyclization, lipidation, and the design of peptidomimetics. Compared to natural AMPs, which are rapidly degraded by proteases and may exhibit cytotoxicity at high concentrations, synthetic peptides demonstrate greater tolerability and sustained activity in biological environments [142,146,147,148]. The development of synthetic AMPs is one of the most active areas in the development of modern antimicrobial drugs, with numerous AMP-based therapeutic candidates currently under pre-clinical and clinical evaluation. Several well-studied molecules, including those known under the names Omiganan, Pexiganan, and Murepavadin, have advanced to Phase II or Phase III clinical trials, and examples of these compounds are presented in the already mentioned Table 1 [149,150,151]. Thus, synthetic antimicrobial peptides extend the anti-infective potential of natural AMPs by combining enhanced stability and potency with targeted activity against multidrug-resistant intestinal pathogens.

#### Design Strategies and Structural Modifications of Synthetic Antimicrobial Peptides

**Figure 5:**
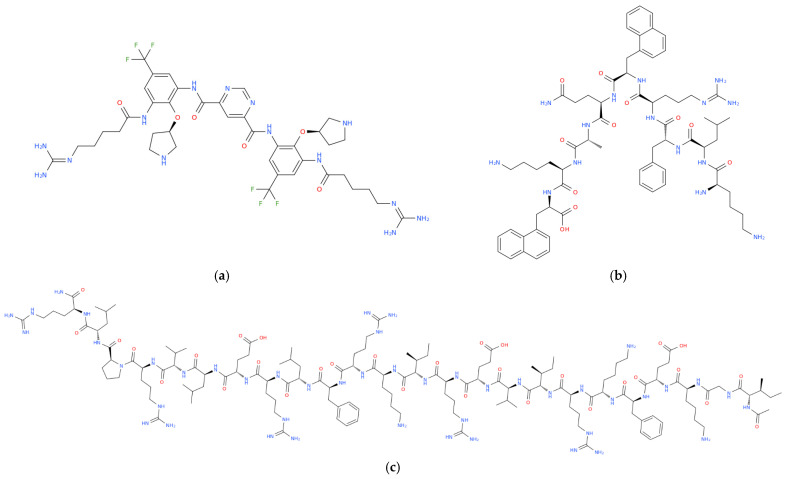
Structural representation of the defensin mimetic brilacidin (**a**), the synthetic peptides XOMA-629 (**b**) and OP-145 (**c**).

Modern strategies for the design of synthetic AMPs rely on rational engineering approaches aimed at increasing their stability, biological activity, selectivity, and pharmacokinetic performance, with structural modifications leading to significantly optimized properties of natural peptides by reducing their toxicity and increasing their efficacy against infections caused by drug-resistant pathogens [12,152,153]. One effective strategy for enhancing peptide stability involves the substitution of L-amino acids with D-amino acids. Because proteases typically recognize and cleave peptides composed of L-amino acids, the incorporation of D-amino acids markedly increases peptide resistance to proteolytic degradation and prolongs their half-life in biological environments. In certain cases, this modification may also reduce immunogenicity. Moreover, D-amino acid substitution can stabilize secondary structures while preserving antimicrobial activity. Notable examples include the D-configured antimicrobial peptide D-IK8 and the D-modified immunoregulatory decapeptide RDP58, both of which exhibit improved stability while maintaining potent biological activity [154,155,156]. Another strategy involves cyclization of the polypeptide chain. Cyclization of AMPs, whether head-to-tail, side-chain-to-side-chain, disulfide bond-mediated, or lasso-like, restricts conformational flexibility, stabilizes the bioactive conformation, and protects the peptide backbone from enzymatic degradation. Consequently, cyclic AMPs frequently demonstrate improved bioavailability, enhanced membrane affinity, and prolonged in vivo activity. Representative examples include brilacidin, a non-peptidic defensin mimetic, and lariocidin, a ribosomally synthesized lasso peptide with exceptional structural stability, both of which exhibit broad-spectrum antibacterial activity [157,158,159]. Another strategy involves lipidation and aromatic enhancement. The attachment of fatty acid chains to the N-terminus or specific side chains of a peptide increases hydrophobicity, thereby strengthening interactions with bacterial membranes and promoting rapid membrane disruption. Lipidated AMPs and lipopeptides often display potent activity against otherwise difficult-to-treat strains, including biofilm-forming and multidrug-resistant bacteria, although their hydrophobicity must be carefully balanced to avoid excessive cytotoxicity. A representative example is HB1345, a short synthetic lipohexapeptide that demonstrates broad-spectrum antimicrobial and anti-inflammatory activity against skin pathogens, including *Cutibacterium acnes*, with relatively low cytotoxicity in pre-clinical models [160,161,162]. Another promising strategy involves the development of peptidomimetics and β- and γ-peptides. Peptidomimetics are fully or partially non-peptidic structures that replicate the key physicochemical properties of natural antimicrobial peptides, including cationic charge, amphiphilicity, and spatial separation of hydrophobic and hydrophilic domains. These molecules exhibit superior metabolic stability and pharmacokinetic profiles compared to natural AMPs. The incorporation of β- and γ-amino acids, as well as aryl-amide foldamers, enables the formation of stable secondary structures such as β-helices or helical foldamers that resist proteolytic degradation. Brilacidin (Figure 5a), a non-peptidic defensin mimetic based on an aryl-amide foldamer, disrupts bacterial membranes and has progressed to Phase II clinical trials.

Similarly, XOMA-629 (Figure 5b), a synthetic peptide derived from bactericidal/permeability-increasing proteins, functions as an AMP mimetic with potent activity against *Staphylococcus aureus* (including MRSA) and has been clinically evaluated for impetigo [163,164,165,166]. Another methodology involves the use of hybrid and chimeric constructs. Hybrid AMPs are generated by combining functional fragments from different natural peptides, thereby integrating complementary activities such as membrane permeabilization, antibiofilm effects, and immunomodulation into a single molecule. In this context, a notable example is OP-145 (Figure 5c), a rationally designed 24-mer peptide derived from the human cathelicidin LL-37, in which the proteolytically unstable *N*-terminal region was removed and the remaining sequence optimized. Compared with its parent peptide, OP-145 exhibits improved stability, a more favorable therapeutic index, and potent activity against pathogens such as methicillin-resistant *Staphylococcus aureus*, while retaining key functional characteristics of LL-37. Collectively, strategies including D-amino acid substitution, cyclization, lipidation, peptidomimetic design (incorporating β- and γ-peptides), and hybrid constructs all contribute to modern AMP engineering, driving the development of candidates with enhanced efficacy and drug-like properties suitable for clinical application [167,168,169,170]. The search for novel therapeutic approaches to address diseases of social importance emphasizes nature-inspired methods [171,172,173,174,175,176], alongside molecular frameworks that integrate both natural and synthetic agents [177,178]. These include peptide- and oligonucleotide-based molecules, as well as hybrid constructs such as nucleopeptides [179,180,181,182,183,184,185,186]. In this context, the targeted synthesis of peptidomimetics and analogues containing non-protein amino acids has become a pivotal strategy to overcome stability issues. The incorporation of specific functional groups, such as thiazole, triazole, or fluorenyl–methoxycarbonyl derivatives, has been shown to significantly enhance biological activity of potential drugs [187,188,189,190,191]. Moreover, advanced synthetic methodologies, including the Michael addition reaction and the use of Ni-complexes, have enabled the creation of novel chiral amino acid derivatives and dipeptides that demonstrate potent antifungal and antibacterial activity against resistant pathogens [192,193]. Investigating the biomolecular interactions of these synthetic derivatives, specifically their binding affinity to DNA and serum albumin, provides further crucial insights into their therapeutic potential and pharmacokinetics [194,195]. In summary, synthetic AMPs are a critical component of modern strategies to combat infections caused by multidrug-resistant microorganisms, and as global antibiotic resistance grows, the demand for agents with alternative mechanisms and reduced susceptibility to resistance development increases. Synthetic AMPs meet these requirements by combining rapid membrane-disrupting activity, immunomodulatory properties, and a relatively low rate of resistance development, particularly when enhanced through rational design and chemical modifications, and their ability to simultaneously target multiple pathogenic pathways is a major advantage. In addition to pore formation and depolarization, many engineered peptides inhibit biofilm formation and eradicate mature biofilms, which is crucial for treating chronic infections associated with *Staphylococcus aureus*, *Pseudomonas aeruginosa*, and *Candida* species, while synthetically optimized molecules can also achieve pathogen-specific activity, opening therapeutic options against highly resistant *P. aeruginosa* strains. Several synthetic AMPs have progressed to advanced clinical evaluation, confirming their translational potential, and their multifaceted mechanisms, reduced risk of resistance compared to conventional antibiotics, biofilm-targeted activity, and synergistic effects with traditional antibiotics support their use as both monotherapy and adjunctive therapy for chronic, nosocomial, and recurrent infections. Moreover, modern drug delivery innovations, including nanoformulations, liposomal carriers, inhaled formulations, and transdermal systems, further enhance different AMP properties such as stability, bioavailability, and tissue targeting, thereby expanding their clinical applicability [196,197,198,199]. Thus, rational design and structural optimization of synthetic antimicrobial peptides enable the development of next-generation intestinal anti-infective agents with improved pharmacological properties and sustained efficacy in the gastrointestinal environment.

## 3. Challenges and Innovations in AMP Development

Antimicrobial peptides are important components of innate immunity, actively participating in the regulation of microbial homeostasis, fighting pathogenic infections, and maintaining the epithelial barrier. More in detail, natural AMPs can disrupt membranes and modify the immune system by neutralizing toxins, reducing inflammation, and accelerating mucosal healing. However, as numerous studies have shown, their clinical use is limited by rapid proteolysis, low stability, and poor bioavailability, particularly in the gastrointestinal tract [15,16,33,200,201]. These limitations have prompted the development of synthetic AMPs that can replicate the key biological effects of natural molecules while being more stable and pharmacologically predictable. Structural and chemical modifications have played a key role in the development of synthetic AMPs, including processes such as the introduction of D-amino acids, the creation of cyclic and lipidated peptides, the design of short cationic molecules, and the formation of hybrid structures and peptidomimetics [16,31,157,202,203]. These approaches have enabled the creation of compounds that are resistant to enzymatic degradation, yet retain activity under conditions of high ionic strength and in the presence of serum, bile, or mucus secretions. The use of these strategies has enabled the development of molecules such as those known under the names Brilacidin, OP-145, Pexiganan, Omiganan, PAC-113, and Murepavadin, which have demonstrated efficacy in Phase II–III clinical trials [40,43,58,61]. It is important to emphasize that progress in the creation of synthetic AMPs has been made possible not only by chemical modifications, such as by the introduction of D-amino acids, cyclization, lipidation, and the creation of hybrid structures, but also by the emergence of large specialized databases containing the sequences, structural parameters, and biological activity of thousands of peptides. In particular, APD (Table 2) offers a well-annotated collection of natural AMPs, while CAMPR4 stands out for its very large dataset and integrated prediction tools. DRAMP provides one of the most comprehensive repositories, including detailed activity and toxicity annotations. BACTIBASE focuses specifically on bacteriocins, and MilkAMP compiles bioactive peptides derived from milk proteins. PhytAMP is dedicated to plant AMPs, whereas AVPdb and HIPdb specialize in antiviral and HIV-inhibiting peptides, respectively. Moreover, DBAASP uniquely links antimicrobial activity with structural data, while Hemolytik is particularly useful for evaluating hemolytic potential and safety profiles of peptides (Table 2). These resources provide a foundation for computer modeling, efficacy prediction, optimal motif selection, and in silico screening prior to laboratory synthesis, significantly accelerating the development of new peptides. Modern development of synthetic AMPs would not be straightforward without the use of global bioinformatics resources (Table 2). International antimicrobial peptide databases accumulate information on over 40,000 natural and synthetic molecules, enabling the systematization of structural motifs, analysis of physicochemical parameters, and in silico efficacy prediction. These databases serve as the basis for machine learning algorithms, 3D modeling, structural docking, and computational design of new peptides, significantly reducing the time and cost of searching for active candidates [204,205,206,207,208].

By performing integrated sequence–structure–activity relationship analyses, researchers can assess peptide toxicity, hydrophobicity, amphiphilicity, charge distribution, and the structural domains that determine antibacterial spectrum. These resources facilitate the rational design of synthetic molecules with high efficacy, low resistance potential, and improved selectivity for therapeutic applications. However, despite significant progress, several challenges remain, including the high cost of peptide synthesis, limited in vivo efficacy, the need for improved pharmacokinetics, potential cytotoxicity at elevated concentrations, and insufficient knowledge of AMP effects on commensal microbiota during long-term treatment [209,210,211]. In this context, nanotechnology-based delivery systems, such as liposomal formulations, solid lipid nanoparticles (SLNs), polymer capsules, and targeted carriers, represent a promising approach to enhance local AMP concentrations, reduce toxicity, and prolong therapeutic activity. In parallel, artificial intelligence and machine learning will contribute to accelerating AMP sequence optimization, thus reducing attrition rates among candidate molecules. Collectively, the advances summarized in this work demonstrate the growing importance of natural and synthetic AMPs as next-generation antimicrobial drug candidates capable of addressing the global rise in antibiotic resistance. Consequently, continued progress in peptide delivery systems will depend on the synergistic integration of computational methods, bioengineering, clinical trials, and emerging technologies [212,213].

### Structure–Activity Relationships and Selectivity of Antimicrobial Peptides

Understanding the structure–activity relationship (SAR) [214] of antimicrobial peptides is essential for designing molecules with potent activity and acceptable safety profiles. Sequence-based templates have proven particularly useful in this regard, as they guide the rational design of new analogues and enable the identification of promising peptide fragments within protein databases. For example, applying a helical template to database searches allowed the discovery of an N-terminal segment of pilosulin 1 that, once synthesized, exhibited broad-spectrum antimicrobial activity [215]. Subsequent targeted substitutions further enhanced antibacterial potency while reducing hemolysis, illustrating how small structural adjustments can markedly improve selectivity. A central determinant of AMP activity is its interaction with lipid membranes. For α-helical peptides, membrane engagement aligns with carpet-like mechanisms of disruption, and experimental correlations between peptide structuring in membrane-mimetic environments and bacterial permeabilization support this model. Features such as hydrophobic–polar face distribution, insertion depth, and the snorkel effect strongly influence efficacy. These same structural properties also shape cytotoxicity toward host cells, as conformational stability and hydrophobicity can shift the balance between antimicrobial potency and safety [215].

Selectivity studies further highlight the importance of SAR-guided design. AMPs differ widely in their ability to discriminate between bacterial membranes and mammalian intestinal cells. For example, gallidermin demonstrates high selectivity, combining strong antimicrobial activity with minimal cytotoxicity and negligible hemolysis [216]. On the other hand, nisin A shows moderate selectivity, remaining safe at antimicrobial concentrations but becoming harmful only at much higher doses. Magainins occupy an intermediate position, displaying more cytotoxicity than gallidermin or nisin but without causing major structural damage to intestinal cells. In contrast, melittin exhibits poor selectivity, as its antimicrobial concentrations overlap with those that induce hemolysis, epithelial barrier disruption, and loss of microvilli. These comparisons reveal that only certain AMPs possess the membrane specificity required for therapeutic use, while others, such as melittin, may be too broadly cytolytic for safe antimicrobial application but could still serve alternative purposes, including drug-delivery enhancement [216].

## 4. Conclusions

Antimicrobial peptides remain one of the most promising classes of molecules for the development of next-generation anti-infective agents, particularly in intestinal disease, where conventional antibiotics are losing efficacy. Natural and synthetic AMPs combine broad activity against multidrug-resistant pathogens with biofilm disruption and immunomodulatory effects, while modern design strategies, such as incorporation of D-amino acids, cyclization, lipidation, and peptidomimetics, have markedly improved their stability, selectivity, and clinical potential. Despite persisting challenges in delivery and pharmacokinetics, recent advances demonstrate steady progress toward clinical translation, supported by innovations in nanotechnology, as well as combination therapy, and personalized medicine. A central theme emerging from this review is the role of antimicrobial peptides as integrated intestinal anti-infective agents that act beyond simple pathogen killing. Both natural and synthetic AMPs contribute to intestinal defense by combining direct antimicrobial activity against enteric pathogens with immunomodulatory and barrier-protective functions. Epithelial peptides such as defensins, cathelicidin LL-37, and RegIII lectins not only restrict the growth and invasion of gastrointestinal pathogens but also regulate host–microbiota interactions, enforce spatial segregation between microbes and the epithelium, and prevent excessive inflammatory responses that exacerbate intestinal disease. At the molecular level, the conserved structural features of natural AMPs enable rapid targeting of fundamental membrane properties, while synthetic peptides further extend this anti-infective potential through enhanced stability, bioavailability, and activity against multidrug-resistant organisms. Together, these properties position antimicrobial peptides as a promising class of next-generation intestinal anti-infective agents capable of addressing both microbial eradication and preservation of intestinal homeostasis. In our opinion, the anti-infective role of short AMP sequences should be more extensively explored, as compact peptides may retain antimicrobial and immunomodulatory activity while offering superior manufacturability, in some cases a reduced toxicity, and easier integration into delivery systems. This novel direction, together with synergistic use alongside traditional antibiotics, positions AMPs not only as adjuncts but as central components of future infection-control strategies in the face of escalating antibiotic resistance.

## Figures and Tables

**Table 1:** Antimicrobial peptides as drug candidates: clinical and pre-clinical overview *.

Name	Sequence/Structural Details	Activity/Mechanism	Phase	Peptide Type	Developer	Refs.
**CZEN-002 (CKPV)_2_**	(CKPV)_2_	Antifungal, immunomodulatory (α-MSH analog)	IIb	Synthetic octapeptide	Zengen	[34,35]
**HB1345**	Decanoyl-KFKWPW	Lipopeptide; membrane disruption	Pre-clinical	Synthetic (lipidated AMP)	Helix BioMedix	[36,37]
**hLF1-11**	GRRRRSVQWCA	Lactoferrin fragment; immune-activating	I/II	Synthetic	AM-Pharma	[38,39]
**IMX-942 (IDR-1)**	KSRIVPAIPVSLL	Innate defense regulator (IDR)	I/II	Synthetic cationic peptide	Inimex	[40,41]
**OP-145**	LL-37 derivative	Anti-biofilm, antibacterial	II	Synthetic	OctoPlus	[40,42]
**PAC-113 (P-113)**	AKRHHGYKRKFH	Anti-Candida (histatin-5 fragment)	IIb	Semi-synthetic	Pacgen	[43,44]
**Brilacidin**	Not disclosed	Defensin-mimetic; membrane disruption	II	Peptidomimetic	Cellceutix/Innovation Pharma	[40,45,46]
**XOMA-629**	KLFR-(d-naphtho-Ala)-QAK-(d-naphtho-Ala)	Antimicrobial activity via an immunomodulatory mechanism	IIa	Peptidomimetic	XOMA	[40,47,48]
**Arenicin-3**	β-sheet AMP	Anti-MDR; membrane-active	Pre-clinical	Natural AMP	Adenium	[49,50]
**RDP58**	D-amino acid decapeptide	TNF-α suppression, anti-inflammatory (immunomodulator)	II	All-D synthetic peptide	Genzyme	[51,52]
**Iseganan (IB-367)**	RGGLCYCRGRFCVCVGR-NH_2_	Protegrin analog; antibacterial	III	Semi-synthetic	Ardea Biosciences	[53,54]
**LTX-109**	Not disclosed	Membrane-active peptidomimetic	II	Peptidomimetic	Lytix Biopharma	[55,56,57]
**Omiganan**	ILRWPWWPWRRK	Indolicidin analog; anti-biofilm	II/III	Semi-synthetic	Cutanea Life Sciences	[58,59,60]
**Pexiganan (MSI-78)**	MSI-78	Pore-forming antimicrobial	III	Semi-synthetic (magainin analog)	Magainin Pharmaceuticals; MacroChem	[61,62]
**Plectasin-212 (NZ2114 class)**	Defensin variant	Lipid II binding	Pre-clinical	Engineered defensin	Novozymes	[63,64,65]
**Murepavadin (POL7080)**	β-peptide	LptD inhibitor (*P. aeruginosa*)	Advanced clinical (systemic use limited by toxicity)	Peptidomimetic	Polyphor	[66,67,68]
**LC-AMP-I1**	GRMQEFIKKLK	Ultra-short AMP; MDR activity	Pre-clinical	Natural-derived (spider venom)	Chongqing University	[69,70,71]
**Lariocidin**	Lasso peptide	Pore-forming antimicrobial	Pre-clinical	Natural RiPP	Spanish research groups	[72,73]
**WR12**	RWKIFKKIEKMGRNIR	Anti-MRSA/VRSA	Pre-clinical	Synthetic de novo peptide	Hong Kong University	[74,75,76]
**D-IK8**	All-D peptide	Protease-resistant AMP; MDR Gram-	Pre-clinical	All-D synthetic	Korean research groups	[74,77]
**ZP2**	Synthetic AMP	Quorum-sensing inhibition	Pre-clinical	Synthetic	Chinese research institutes	[78,79,80]
**Glutoxim**	γ-glutamyl-cysteine	Immunomodulator	Marketed	Synthetic (glutathione analog)	Pharmasyntez	[81,82]
**Acipensin-1**	Fish-derived AMP	Membrane lysis	Pre-clinical	Natural AMP	Far East Biomedical Labs	[83,84]

* Clinical development stages reported in this table refer to the most advanced phase reached; in some cases, systemic development was limited or discontinued due to safety concerns, while alternative formulations remain under investigation.

**Table 2:** Major antimicrobial peptide databases: comprehensive repositories of natural and synthetic AMPs.

Database	Dataset Size *	Content	Website **
APD	2619	Natural and some synthetic AMPs	https://aps.unmc.edu/AP/
CAMP_R4_	Natural AMPs: 11,827 Synthetic AMPs: 12,416	Collection of antimicrobial peptides	https://camp.bicnirrh.res.in/
DRAMP	30,260	Data repository of antimicrobial peptides	http://dramp.cpu-bioinfor.org/
BACTIBASE	177	Bacteriocins	http://bactibase.pfba-lab.org
MilkAMP	371	Milk bioactive peptide database	http://mbpdb.nws.oregonstate.edu/
PhytAMP	273	Plant AMPs	https://phytamp.hammamilab.org/entrieslist.php
AVPdb	2683	Database of antiviral peptides	http://crdd.osdd.net/servers/avpdb/
HIPdb	981	Database of experimentally validated HIV inhibiting peptides.	http://crdd.osdd.net/servers/hipdb/index.php
DBAASP	~8000	Database of antimicrobial activity and structure of peptides	https://dbaasp.org/home
Hemolytik	~5000	Database of hemolytic and non-hemolytic peptides	http://crdd.osdd.net/raghava/hemolytik/

* Reported dataset sizes are approximate and reflect the number of entries available at the time of access (December 2025); database content is subject to continuous updates and may vary depending on inclusion criteria. ** All links displayed in this table were accessed on 18 December 2025.

## Data Availability

No new data were created or analyzed in this study.

## Abbreviations

The following abbreviations are used in this manuscript:

Abbreviation	Full name/Meaning
AMP	Antimicrobial Peptide
CAMP	Cationic Antimicrobial Peptide
HD-5/HD-6	Human Defensin 5/6
LL-37	Human Cathelicidin Peptide
HBD	Human Beta-Defensin
MDR	Multidrug-Resistant
XDR	Extensively Drug-Resistant
MRSA	Methicillin-Resistant Staphylococcus aureus
SSTI	Skin and Soft Tissue Infections
LPS	Lipopolysaccharide
LTA	Lipoteichoic Acid
QS	Quorum Sensing
GI Tract	Gastrointestinal Tract
IBD	Inflammatory Bowel Disease
NEC	Necrotizing Enterocolitis
PRR	Pattern-Recognition Receptor
TLR	Toll-Like Receptor
NF-κB	Nuclear Factor kappa B
MAPK	Mitogen-Activated Protein Kinase
CFU	Colony-Forming Unit
MIC	Minimum Inhibitory Concentration
IDR	Innate Defense Regulator
PAMPs	Pathogen-Associated Molecular Patterns
APC	Antigen-Presenting Cell
IECs	Intestinal Epithelial Cells
SCFAs	Short-Chain Fatty Acids
ROS	Reactive Oxygen Species
